# Dorso-medial prefrontal cortex responses to social smiles predict sociability in early human development

**DOI:** 10.1162/imag_a_00129

**Published:** 2024-04-08

**Authors:** Tobias Grossmann, Olivia Allison

**Affiliations:** Department of Psychology, University of Virginia, Charlottesville, VA, United States

**Keywords:** brain, social cognition, social behavior, infancy, prefrontal cortex, fNIRS

## Abstract

Dorso-medial prefrontal cortex (dmPFC) plays a vital role in social cognition and behavior among humans. Enhanced responses in dmPFC when viewing social scenes predict increased levels of sociability in adults. The current longitudinal study examined the association between dmPFC response and sociability in early development. Brain responses were measured in response to social smiles and frowns using functional near-infrared spectroscopy (fNIRS) at 11 months. Individual differences in sociability were measured using the Early Childhood Behavior Questionnaire (ECBQ) at 18 months. Our longitudinal results show that greater engagement of the dmPFC when processing social smiles, but not frowns, at 11 months predicts higher levels of sociability at 18 months. This demonstrates that early variability in dmPFC response during positive social interactions is linked to individual differences in overtly displayed social behavior. This supports the view that dmPFC plays an important role in social cognition and behavior from early in human ontogeny.

## 1 Introduction

Humans are ultra-social animals who live in complex groups (Tomasello, 2014,^2019^). One of the most essential skills in navigating our social environments is our ability to identify friendly (prosocial) individuals that we can affiliate and cooperate with and distinguish them from unfriendly or even hostile (antisocial) individuals that we may want to avoid (Fiske & Taylor, 1991). In fact, according to the self-domestication hypothesis, human evolution is thought to be characterized by positive selection for friendliness traits, fostering human-unique theory of mind and prosocialty (Hare, 2017). The human capacities for theory of mind and prosociality have deep ontogenetic roots, as they have been shown to develop early during infancy (Grossmann, 2017;Hamlin et al., 2007;Kovács et al., 2010;Warneken, 2015). In studies using functional magnetic brain imaging (fMRI) with human adults, the dorso-medial prefrontal cortex (dmPFC) has been identified as a key brain region involved in social cognitive processes critical for theory of mind and prosociality (Amodio & Frith, 2006;Waytz et al., 2012).

A recent study tested the hypothesis that from early in development humans rely on processes localized in dmPFC when interacting with, and socially evaluating, friendly and threatening individuals (Krol & Grossmann, 2020). This study shows that infants, like adults in previous studies (Mende-Siedlecki et al., 2013;Mitchell et al., 2005,^2006^), rely on processes localized in dmPFC during social evaluation. Specifically, infants’ dmPFC responses distinguish between friendly and threatening individuals during eye contact and predict their personal preferences during social evaluation (Krol & Grossmann, 2020). These findings demonstrate that the dmPFC is involved in social evaluation in human infants, providing evidence that the brain system supporting person perception develops early in human ontogeny. This suggests that the ability to socially evaluate others during social encounters may represent a foundational element of human social cognition. It is important to mention that there is a growing body of evidence showing that the dmPFC role extends beyond social evaluation in infancy and, more broadly, includes functions pertaining to the interpretation of social eye cues (seeGrossmann, 2017, for review), also during live social interactions (Urakawa et al., 2015). However, to date, little is known about what role dmPFC plays in guiding overt social behavior during early development.

Among adults, individuals who display greater activity in dmPFC when viewing social scenes spent more time around other people, on a daily basis, suggesting that dmPFC response during social information processing links to individual differences in real-life sociability (Powers et al., 2016). The current study longitudinally assessed whether variability in infants’ dmPFC response during social evaluation at 11 months of age predicts social behavior, especially sociability levels, at 18 months of age. Sociability was defined as observed positive social-interactive behaviors reflected in actively seeking out social contact and taking pleasure in interactions with others (Putnam et al., 2006). Our pre-registered hypothesis (https://osf.io/zpwh4) was that dmPFC responses longitudinally predict sociability levels, with greater dmPFC responses to social smiles (individuals displaying positive social-interactive behaviors) being positively predictive of heightened levels of sociability (positive social-interactive behaviors reflected in actively seeking out social contact and taking pleasure in interactions with others).

## 2 Materials and Methods

### 2.1 Participants

76 typically developing infants (38 females) participated in this study (11 months fNIRS data:*M_age_*= 339.94 days,*SE*= 0.744; 18 months ECBQ data:*M_age_*= 555.07 days,*SE*= 1.448). This study represents a pre-registered reanalysis (https://osf.io/zpwh4) of existing fNIRS data from 11-month-old infants (Krol & Grossmann, 2020) and is part of a larger longitudinal study of infant social, cognitive, and brain development (seeGrossmann et al., 2018). All infants were born at normal birth weight (>2,500 grams) and at standard gestational age (>38 weeks). Infants were of parent-reported European descent. From the full sample of seventy-seven typically developing 11-month-old infants, 69 infants were included in the final analysis (n = 8 failed to either complete the ECBQ [n = 3] or provide usable fNIRS data [n = 5]). This study was approved by the Ethics Committee at the Medical Faculty, Leipzig University (236-10-23082010) and was conducted in accordance with the Declaration of Helsinki. Parents provided written informed consent and were compensated with travel money, a toy for the infant, and a printed photograph of their infant in the fNIRS cap.

### 2.2 Stimuli

Color photographs of Caucasian females with direct-gaze expressions of happiness (displaying smiles), anger (displaying frowns), and neutrality were chosen from a validated stimulus set (FACES Collection) (Ebner et al., 2010). Four actress identities were selected based on expression recognition rates by groups of young, middle-aged, and older adults as well as on the basis of minimal distracting features (i.e., hair was not obstructing face). Average expression recognition accuracy within the four selected identities was over 94.92% (seeEbner et al., 2010). Eye gaze was manipulated using Adobe Photoshop CS5 for use in the fNIRS paradigm. Photographs were resized and cropped to align with fixed markers for the position of the two eyes, mouth, and nose in the center of a gray background. This editing technique ensured that all facial features were presented in the same location on the screen. Baseline images consisted of color photographs of four inanimate objects (vegetables) presented in the center of the same light gray background. These images have been successfully used as baseline stimuli in fNIRS studies of face processing in infants (Grossmann et al., 2018;Krol et al., 2019;Nakato et al., 2009,^2011^;Otsuka et al., 2007).

### 2.3 Procedure

Infants were seated on a parent’s lap in a quiet, dimly lit room, facing a screen (52 cm x 32 cm) at approximately 60 cm. A room divider separated the experimental area from the control desk, and a black curtain covered the region behind the presentation monitor to prevent distractions. As in prior studies (Grossmann et al., 2018;Krol et al., 2019), a plastic ring attached to the chair was provided for each infant to hold to reduce arm and body movements. A camera was attached to the bottom of the screen for online tracking of infant behavior as well as offline coding of attention to each trial.

The fNIRS paradigm consisted of blocks of four randomized trials of smiling-direct, smiling-averted, frowning-direct, and frowning-averted facial expressions. Critically, each of the four identities consistently presented the same expression-gaze combination throughout the experiment. A total of 24 different identity-expression-gaze combination scenarios were created and were counterbalanced across infants. Each presentation block began with an attention-getter to keep infants alert and to orient them to the center of the screen (a shaking rattle, as described inKrol et al., 2015). Each trial began with presentation of a baseline stimulus for at least six seconds followed by a face stimulus for six seconds. At the beginning of each baseline and face presentation (twice per trial), a brief 150-millisecond bell tone (about 600 Hz) sounded to maintain infant attention. Baseline and face stimuli were presented dynamically. The baseline shifted from an image changing from its original size (500 ms) to a slightly larger size (~1° increase in visual angle) (700 ms) at least five times. Face presentation consisted of three photographs of the same identity: a) a neutral expression with the non-target gaze (250 ms), b) a neutral expression with the target gaze (250 ms), and finally, c) the target expression (smile or frown) with the target gaze (direct or averted) (700 ms). This sequence repeated five times to create the illusion of a neutral individual first dynamically changing their gaze and subsequently changing their expression from neutral to either a smile or frown (Krol & Grossmann, 2020). This method of pseudo-dynamic presentation of facial expressions was adapted from previous infant fNIRS paradigms (Grossmann et al., 2018;Krol et al., 2019;Nakato et al., 2011), ensuring that infants kept attention during the long trials that fNIRS measurement requires. Stimulus presentation was counterbalanced such that no expression or gaze trial type was shown more than twice in succession. Infants were shown an average of 25.65 total fNIRS trials (*range*= 10 to 46;*SD*= 7.57).

Stimuli were presented using Presentation software (Neurobehavioral Systems, MA), and fNIRS data were recorded using an NIRScout system and NIRStar acquisition software (NIRx, Berlin, Germany). Hemoglobin absorption was measured using 32 optodes (16 sources, 16 detectors) placed at approximately 2 cm distance over frontal and temporal cortices on a custom-built elastic cap (EasyCap, Germany) with standard 10–20 references. This arrangement comprised 49 channels (source-detector pairs) from which to measure hemodynamic change. Data were recorded at a sampling rate of 6.25 Hz. Near-infrared light was emitted at two wavelengths (760 nm and 850 nm) with a power of 5 nm/wavelength. The system automatically adjusted light intensity to provide optimal gain.

### 2.4 fNIRS analysis

Videos were recorded to monitor infants’ attention to the screen on which the stimuli were presented to allow for off-line coding of infant attention to the stimuli. Based on these video recordings, each session was manually coded off-line for infant looking duration to each trial. As a pre-determined criterion, trials were only included if infants continuously attended to the screen for at least four seconds (2/3 of the stimulus duration) of the 6 seconds for which stimuli were presented. The fNIRS data were then visually inspected for motion artifacts. Trials with motion artifacts were removed from further analyses. The remaining data were analyzed using the Matlab-based software Nilab2 (NIRx, Germany). Data were filtered with a 0.2-Hz low-pass filter to remove fluctuations due to infant heart rate and a high-pass filter of 12 seconds to remove changes too slow to be related to experimental stimuli (i.e., fluctuations due to drift). Measurements were converted into oxygenated hemoglobin (oxy-Hb) and deoxygenated hemoglobin (deoxy-Hb) using the modified Beer-Lambert law. Boxcar functions corresponding to the four stimulus conditions were convolved with a standard hemodynamic response function based on the stimulus length parameter (Boynton et al., 1996). The average concentration changes of oxy-Hb and deoxy-Hb in response to each stimulus condition were extracted for each channel, for each individual infant. Only infants who provided at least two artifact-free trials per condition were included in fNIRS analyses. The region of interest (ROI) of the dorso-medial prefrontal cortex (dmPFC) was created by referencing anatomical sources of the infant 10–20 system (Kabdebon et al., 2014) and through the use of nirsLAB and NIRSite software (NIRx), which projects fNIRS channels onto MNI space (locked to 10–20 coordinates) (seeFig. 1). From the average concentration change for each of the four conditions, two difference scores were computed to determine dmPFC response to social smiles (direct gaze smile minus averted gaze smile) versus social frowns (direct gaze frown minus averted gaze frown). Note that mPFC ROI used inKrol and Grossmann (2020)overlaps with the dmPFC ROI used in the current study for two out of the three channels used. The one channel that differs between studies is now located more dorsally to best capture responses from dmPFC and allow for comparability between the current study andPowers et al.’s (2016)study with adults, which served as the basis for the current study with infants. An additional ROI was created to measure responses from the ventro-medial prefrontal cortex (vmPFC) as a control region (seeFig. 1) in order to examine whether any effects are specific to dmPFC, as seen in prior work using fMRI with adults (Powers et al., 2016).

**Fig. 1.**
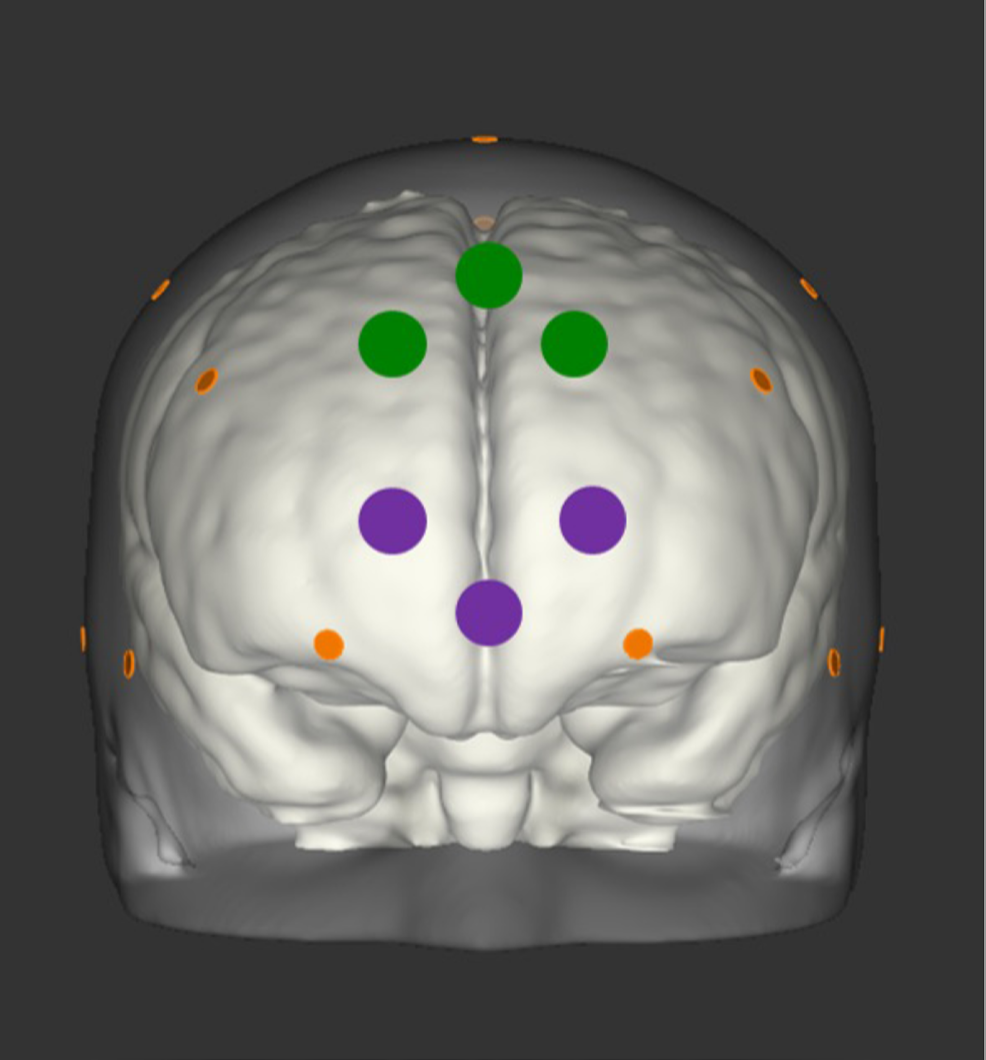
This figure shows the fNIRS channel mapping onto the cortical surface (frontal view) for the dmPFC ROI (in red) and the vmPFC ROI (in blue) with reference to the 10-20 electrode placement system (in orange).

### 2.5 Sociability

Individual differences in sociability—defined as behaviors indicative of seeking and taking pleasure in interactions with others—were measured using the sociability scale taken from the commonly used and validated Early Childhood Behavior Questionnaire, ECBQ (Putnam et al., 2006). Children’s sociability levels were determined on the basis of eight parent-reported items (seeTable 1). Parents were prompted to read each item (description of the child’s behavior) and then asked to indicate how often the child did engage in this behavior during the last two weeks by circling one out of eight possible responses. The responses to these items were measured on a Likert scale (ranging from 1 = never, 2 = very rarely, 3 = less than half the time, 4 = about half the time, 5 = more than half the time, 6 = almost always, 7 = always, does not apply = NA). The sociability score for each participating child was determined by computing the mean average of responses to the eight sociability items.

**Table 1.** This table lists that eight items taken from the ECBQ used to determine the sociability score in the current study.

**When a familiar child came to your home, how often did your child** 21. engage in an activity with the child? 22. seek out the company of the child? **When visiting the home of a familiar adult, such as a relative or friend, how often did your child** 40. want to interact with the adult? **When visiting the home of a familiar child, how often did your child** 84. engage in an activity with the child? 85. seek out the company of the child? **When a familiar adult, such as a relative or friend, visited your home, how often did your child** 172. want to interact with the adult? **When around large gatherings of familiar adults or children, how often did your child** 199. want to be involved in a group activity? 200. enjoy playing with a number of different people?

Note that the ECBQ and its scales were not developed to carry out individual item analysis.

## 3 Results

We longitudinally assessed the pre-registered hypothesis (https://osf.io/zpwh4) that infants’ dmPFC responses to social smiles at 11 months of age positively predict sociability levels at 18 months of age. This was done by running a multiple linear regression analysis^1^(entry-method) in IBM SPSS Statistics (Version 28; data and analysis output are available here:https://osf.io/4w9m6), employing three models, entered in three blocks, all with sociability levels at 18 months as the dependent (outcome) variable (seeTable 2, for model summaries and statistics). Model 1 used only infants’ dmPFC responses to social smiles at 11 months as the independent (predictor) variable. Model 2 used infants’ dmPFC responses to social smiles and frowns at 11 months as the two predictor variables. Model 3 used infants’ dmPFC responses to social smiles and frowns at 11 months and infants’ vmPFC responses to social smiles and frowns at 11 months as the four predictor variables. Model 1 revealed a significant effect,*F*(1, 68) = 3.996,*p*= 0.050,*R^2^*= 0.056. Confirming our pre-registered hypothesis, the regression showed that infants’ enhanced dmPFC reponses to social smiles (*ß*= 0.237,*t*= 1.999,*p*= 0.050) were positively associated with infants’ sociability levels (seeFig. 2). The*p-*value obtained for the positive association is exactly at 0.05 but not below the common threshold (<0.05). However, it should also be taken into account that this analysis tested a pre-registered directional hypothesis partly based on prior work using fMRI with adults (Powers et al., 2016). As shown inTable 2, no such effect was seen when dmPFC responses to frowns or vmPFC responses to smiles or frowns were used as predictor variables.

**Fig. 2.**
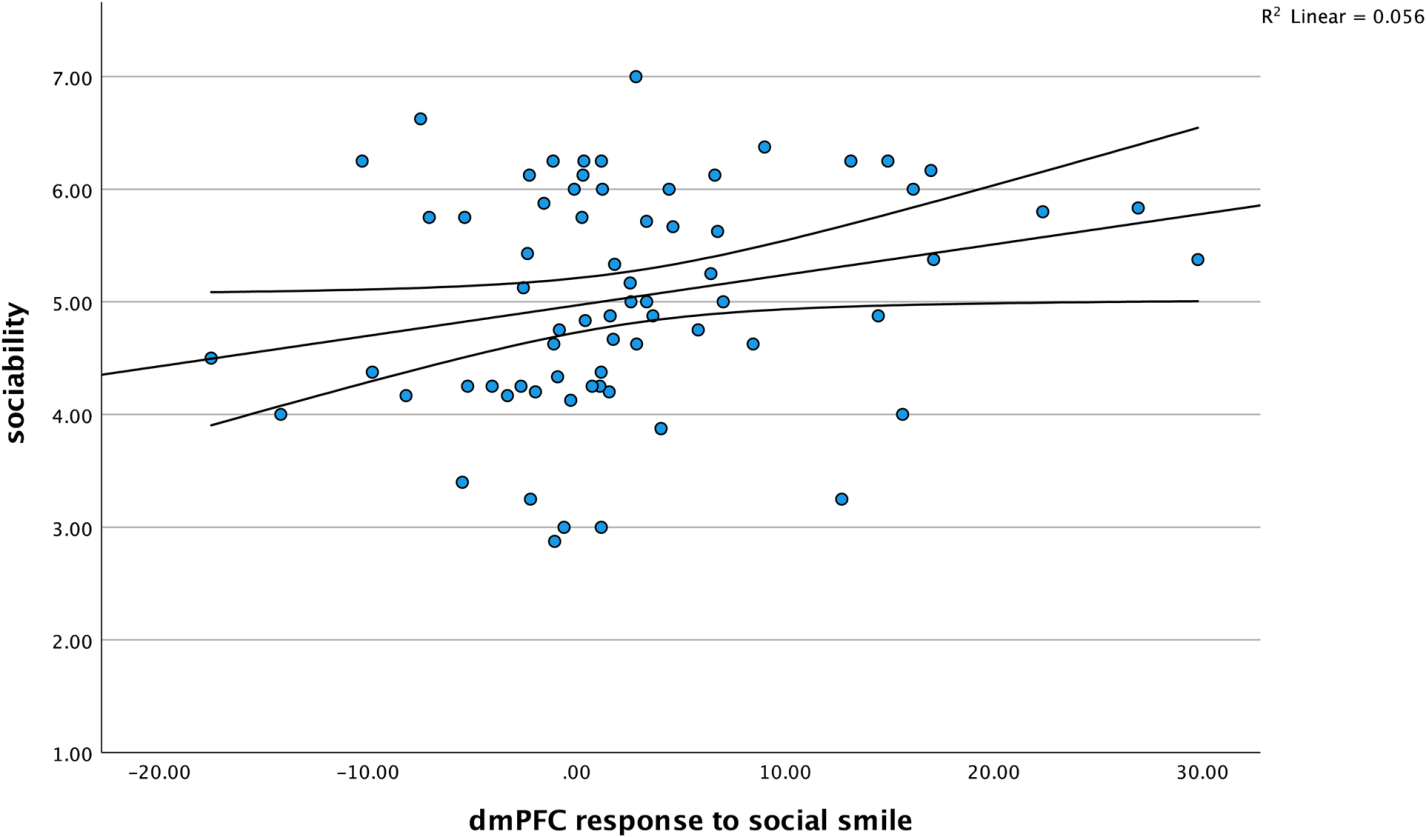
Scatter plot showing dmPFC response (direct gaze smile minus averted gaze smile at 11 months, using oxygenated hemoglobin in micromolar) and sociability levels at 18 months.

**Table 2.** Summary statistics of linear regression analysis, employing three models, entered in three blocks, with sociability levels at 18 months as the dependent variable and using brain responses in dmPFC and vmPFC at 11 months as predictors.

					**Change statistics**				
**Model**	**R**	**R square**	**Adjusted R square**	**Std. error of the estimate**	**R square change**	**F change**	**df1**	**df2**	**Sig. F change**
1	.237 ^a^	.056	.042	.96194	.056	3.996	1	67	.050
2	.239 ^b^	.057	.029	.96869	.001	.069	1	66	.794
3	.246 ^c^	.061	.002	.98191	.003	.117	2	64	.889

**Model predictors**					**Beta**		***t***		***p-*** **value**
1	dmPFC smile				.237		1.999		.050
2	dmPFC smile				.236		1.976		.052
	dmPFC frown				.031		.263		.794
3	dmPFC smile				.225		1.825		.073
	dmPFC frown				.028		.217		.829
	vmPFC smile				-.006		-.043		.966
	vmPFC frown				.059		.470		.640

In an additional analysis, we employed a Bayesian regression analysis in JASP (Version 0.18.3). Similar to the results using the linear regression approach reported above, this Bayesian analysis revealed a BF10 = 1.333 for infants’ dmPFC responses to smiles at 11 months in predicting sociability at 18 months, providing evidence for the pre-registered hypothesis versus the null model. Moreover, when the other predictor variables (dmPFC responses to frowns, vmPFC responses to smiles, vmPFC responses to frowns) were added, dmPFC responses to smiles is the only predictor variable where the BF10 remains above 1 (seeSupplementary Table 1).

## 4 Discussion

The current longitudinal study examined the association between mPFC responses and sociability in infancy. Medial prefrontal cortex responses were measured in a pseudo-dynamic face-to-face engagement procedure, presenting social smiles and frowns using functional near-infrared spectroscopy (fNIRS) at 11 months. Individual differences in sociability were measured using the ECBQ at 18 months. Our longitudinal results showed that greater engagement of the dmPFC when processing social smiles at 11 months predicted higher levels of sociability at 18 months. This confirms our pre-registered hypothesis, demonstrating that neural variability in dmPFC response during positive social engagement is linked to positive social behavioral variability during infancy. This supports the view that variability in dorso-medial prefrontal cortex plays a role in accounting for individual differences in social behavior from early in human ontogeny.

Our finding that enhanced engagement of the dmPFC at 11 months predicted higher levels of sociability at 18 months is in line with prior work with adults showing that individuals who display greater activity in dmPFC when viewing social scenes spent more time around other people on a daily basis (Powers et al., 2016). The current longitudinal study goes beyond previous cross-sectional work with adults by showing that variability in social cognition reflected in dmPFC responses in infancy is longitudinally associated with individual differences in social behavior observable in toddlerhood. In conjunction with prior work, the current results thus suggest that across development increased recruitment of dorso-medial prefrontal brain systems during social information processing systematically links to heightened sociability in a trait-like manner. Given the correlational nature of the current findings, it is not possible to determine whether variability in dmPFC function drives individual differences in sociability or vice versa. Relatedly, it is currently unclear what role early social experience and learning may play in accounting for the observed link.

More generally, these findings provide developmental evidence for the notion that the ability to identify friendly (prosocial) individuals represents a key social cognitive skill (Fiske & Taylor, 1991), which relies upon brain systems implicated in theory of mind and prosocial behavior (Amodio & Frith, 2006;Waytz et al., 2012). This early ontogenetic emergence lends credence to the view that, as stipulated by the self-domestication hypothesis, enhanced friendliness traits, fostering the human-unique theory of mind and prosocial behavior, have played an important role over the course of human evolution (Hare, 2017). The obtained pattern of findings also supports the increasing body of evidence attesting that the brain’s capacities for theory of mind and prosociality are precocious in humans (Grossmann, 2017;Hamlin et al., 2007;Kovács et al., 2010;Warneken, 2015).

The current findings further suggest that enhanced dmPFC engagement during social smiles is associated with higher levels of social motivation and reward, considering that higher levels of sociability in toddlerhood were characterized by greater seeking of and taking pleasure in interactions with others. To observe such a link provides developmental support for theories assigning a critical role to motivational processes in human social cognition (Anderson, 2016;Braver et al., 2014;Chevallier et al., 2013). It is important to acknowledge that the current study design only included a measure characterizing social motivation in toddlerhood (at 18 months) but not in infancy (at 11 months). It is therefore not possible to investigate whether this link is already present in infancy. Future research would thus benefit from including measures of social motivation and investigate its relation to dmPFC responses during social evaluation in infancy (Hepach et al., 2013).

The current study pursued a developmental social neuroscience approach to further our understanding of how variability in brain function is linked to social behavior during early human development. The obtained evidence attests that variability of dmPFC function in infancy is linked to differences in overtly displayed social behavior in toddlerhood. These findings provide novel developmental insights into how brain and behavioral process are linked in the service of human social functioning using a longitudinal approach.

## Supplementary Material

Supplementary Material

## Data Availability

Data and code have been posted on OSF and are publicly available at this link:https://osf.io/q5p3j/.

## Footnotes

1 On the advice of the editor and reviewers, to increase statistical rigor, our analysis method differed slightly from the pre-registered version (entry instead of stepwise approach). Moreover, the genetic analysis noted in the pre-registration was not germane to the present question and will be reported separately.
