# Supplementary material for: Dorso-medial prefrontal cortex responses to social smiles predict sociability in early human development
Source: Imaging Neurosci (Camb). 2024 Apr 8;2:imag-2-00129. doi: 10.1162/imag_a_00129 (PMC12247620; doi:10.1162/imag_a_00129)
Supplement: Supplementary Material [file imag_a_00129-supp.pdf]

Model Comparison - ECBQ_sociability_18mo

Models	P(M)	P(M|data)	BF_M	BF_{10}	R^2
Null model	0.200	0.483	3.744	1.000	0.000
dmPFC_smile_11mo	0.050	0.161	3.648	1.333	0.056
dmPFC_smile_11mo + dmPFC_frown_11mo + vmPFC_smile_11mo + vmPFC_frown_11mo	0.200	0.042	0.175	0.087	0.061
vmPFC_frown_11mo	0.050	0.040	0.800	0.334	0.010
dmPFC_smile_11mo + vmPFC_frown_11mo	0.033	0.039	1.178	0.484	0.060
dmPFC_smile_11mo + dmPFC_frown_11mo	0.033	0.036	1.083	0.447	0.057
dmPFC_smile_11mo + vmPFC_smile_11mo	0.033	0.035	1.052	0.434	0.056
dmPFC_frown_11mo	0.050	0.031	0.612	0.258	0.002
vmPFC_smile_11mo	0.050	0.030	0.597	0.252	0.001
dmPFC_smile_11mo + dmPFC_frown_11mo + vmPFC_frown_11mo	0.050	0.024	0.457	0.194	0.061

Note. Table displays only a subset of models.

Supplementary Table 1. This table shows the results of a Bayesian Regression using the dmPFC and vmPFC smile and frown brain variables at 11 months as predictors and sociability at 18 months as the dependent variable.
